# Human T-lymphotropic virus type-1 p30 alters cell cycle G2 regulation of T lymphocytes to enhance cell survival

**DOI:** 10.1186/1742-4690-4-49

**Published:** 2007-07-16

**Authors:** Antara Datta, Lee Silverman, Andrew J Phipps, Hajime Hiraragi, Lee Ratner, Michael D Lairmore

**Affiliations:** 1 Center for Retrovirus Research, The Ohio State University, Columbus, Ohio, USA; 2 Department of Veterinary Biosciences, The Ohio State University, Columbus, Ohio, USA; 3 Ohio State Biochemistry Program, The Ohio State University, Columbus, Ohio, USA; 4 Drug Safety and Disposition, Millenium Pharmaceuticals, Inc., 45 Sidney Street, Cambridge, Massachusetts, USA; 5 Genentech, Inc. MS68, 1 DNA Way, South San Francisco, California, USA; 6 Department of Medicine, Pathology, and Molecular Microbiology, Washington University School of Medicine, St. Louis, Missouri, USA; 7 Comprehensive Cancer Center, Arthur G. James Cancer Hospital and Solove Research Institute, The Ohio State University, Columbus, Ohio, USA

## Abstract

**Background:**

Human T-lymphotropic virus type-1 (HTLV-1) causes adult T-cell leukemia/lymphoma and is linked to a number of lymphocyte-mediated disorders. HTLV-1 contains both regulatory and accessory genes in four pX open reading frames. pX ORF-II encodes two proteins, p13 and p30, whose roles are still being defined in the virus life cycle and in HTLV-1 virus-host cell interactions. Proviral clones of HTLV-1 with pX ORF-II mutations diminish the ability of the virus to maintain viral loads *in vivo*. p30 expressed exogenously differentially modulates CREB and Tax-responsive element-mediated transcription through its interaction with CREB-binding protein/p300 and while acting as a repressor of many genes including Tax, in part by blocking tax/rex RNA nuclear export, selectively enhances key gene pathways involved in T-cell signaling/activation.

**Results:**

Herein, we analyzed the role of p30 in cell cycle regulation. Jurkat T-cells transduced with a p30 expressing lentivirus vector accumulated in the G2-M phase of cell cycle. We then analyzed key proteins involved in G2-M checkpoint activation. p30 expression in Jurkat T-cells resulted in an increase in phosphorylation at serine 216 of nuclear cell division cycle 25C (Cdc25C), had enhanced checkpoint kinase 1 (Chk1) serine 345 phosphorylation, reduced expression of polo-like kinase 1 (PLK1), diminished phosphorylation of PLK1 at tyrosine 210 and reduced phosphorylation of Cdc25C at serine 198. Finally, primary human lymphocyte derived cell lines immortalized by a HTLV-1 proviral clone defective in p30 expression were more susceptible to camptothecin induced apoptosis. Collectively these data are consistent with a cell survival role of p30 against genotoxic insults to HTLV-1 infected lymphocytes.

**Conclusion:**

Collectively, our data are the first to indicate that HTLV-1 p30 expression results in activation of the G2-M cell cycle checkpoint, events that would promote early viral spread and T-cell survival.

## Background

Human T lymphotrophic virus type 1 (HTLV-1) is the etiological agent of adult T cell leukemia/lymphoma (ATL), which in its acute form is a highly aggressive CD4+ T-cell cancer that is refractory to standard therapies (reviewed in [1-3]). As a complex retrovirus, the HTLV-1 genome encodes structural, enzymatic, regulatory and accessory proteins[2,4]. The pX region of the virus contains four open reading frames (ORFs). ORFs III and IV encode the well characterized Rex and Tax proteins, respectively. Tax is a 40 kDa nuclear phosphoprotein that increases viral transcription from the HTLV-1 LTR (reviewed in [5-7]). The ability of HTLV-1 to cause T-cell transformation is linked to deregulation of cellular gene expression and cell cycle checkpoints by Tax [5]. Rex is a 27 kDa nucleolar phosphoprotein that increases the cytoplasmic accumulation of non-spliced and singly spliced viral RNA (reviewed in [8]). In contrast to the extensive knowledge about the structure and function of Tax and Rex, less is known about the role of pX ORF I and II-encoded proteins in the replication cycle and pathogenesis of HTLV-1.

HTLV-1 p30 is a 241 amino acid nuclear localizing protein encoded by pX ORFII [9], that contains serine and threonine-rich regions with partial homology to the POU family of transcription factors [10]. pX ORFs II mRNA is present in infected cell lines and freshly isolated cells from HTLV-1-infected subjects [11] and in ATL and HAM/TSP patients [12]. Infected human subjects form antibodies [13] and cytotoxic T cells [14] against recombinant proteins or peptides of pX ORF II proteins, confirming the expression of the proteins in HTLV-1 in both disease patients and asymptomatic subjects. Freshly cultured transformed lymphocytes from HTLV-1 patients express both Tax and p30 [15]. Our studies were the first to demonstrated that pX ORF II encoding p30 is necessary for establishment and maintenance of HTLV-1 infection in a rabbit model [16,17]. Emerging evidence indicates that p30 has important roles in the viral and cellular gene expression at both the transcriptional and the post translational level [18-27]. Two recent studies indicate that p30 interacts with Rex and co-localize in nucleolar compartments [27,28]. We have demonstrated that p30 also differentially regulates CREB responsive element and Tax responsive element mediated transcription by interacting with CREB binding protein p300[24,26]. Our microarray studies indicated that p30 is actually a selective repressor of genes including some encoding cell cycle control proteins, while sparing T-cell signaling pathways [25]. Consistent with these findings, a recent study indicated that p30 has the ability to enhance Myc-associated transforming activities and increase S-phase cell cycle progression through its interactions with both Myc and the 60 kDa Tat-interacting protein (TIP-60) [15]. Collectively these studies support the role of p30 as a multi-functional protein with transcriptional and post-transcriptional activities that balances the influence of Tax to regulate viral gene expression and modulates the transcriptional control of the cell cycle.

Transition through the G2/M checkpoint in mammalian cells is strictly controlled by coordinated phosphorylation and dephosphorylation events [29,30]. Cdc25C catalyzes the onset of mitosis [31], but its activity is strictly regulated throughout the cell cycle through differential phosphorylation [32]. Phosphorylation of Cdc25C at serine 216 is mediated primarily by check point kinases 1 and 2 (Chk1 and Chk2) [33], which are activated upon DNA damage resulting in enhanced phosphorylation of Cdc25C at serine 216 and G2 arrest [34-37]. The activity of Cdc25C is increased during the G2-M phase of the cell cycle by hyperphosphorylation of Cdc25C catalyzed by both Cdc2 and PLK [38-41].

Herein, we report that expression of p30 in Jurkat T-cells results in an accumulation of cells in the G2 phase of cell cycle. Our data indicates that expression of HTLV-1 p30 resulted in an increase in phosphorylation of Cdc25C at serine 216 and enhanced nuclear localization of phosphorylated Cdc25C at serine 216. Furthermore, the activated form of Chk1 phosphorylated at serine 345 was increased in p30 expressing Jurkat T-cells. p30 expression was also associated with a decrease in expression of PLK1 and diminished phosphorylation of PLK1 at tyrosine 210. Consistent with less PLK1, p30 expression resulted in reduced phosphorylation of Cdc25C at S-198. Finally, primary human lymphocyte derived cell lines immortalized by an HTLV-1 proviral clone defective in p30 expression were more susceptible to camptothecin induced apoptosis. Collectively, our data indicate that HTLV-1 p30 expression modulates regulatory cell cycle control in T-cells to enhance early viral spread and prolong cell survival.

## Results

**Figure 1:**
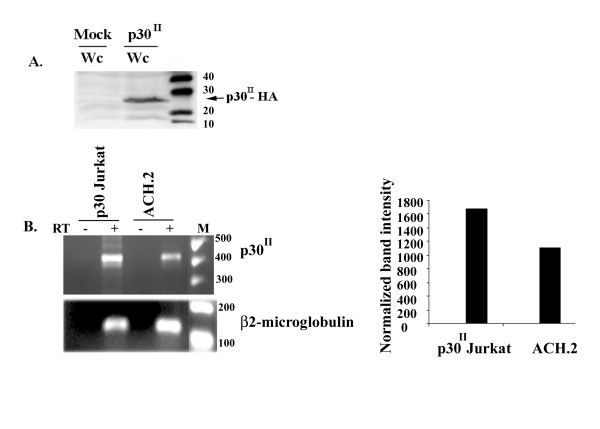
**Expression of p30 in Jurkat T-cells**. A) 40 μg of whole cell extract (Wc) prepared from mock or p30 transduced Jurkat T-cells was loaded on a 10% SDS PAGE gel and analyzed using anti-HA antibody. B) Semi-quantitative RT-PCR: A comparison of p30 mRNA levels in p30 transduced Jurkat T-cells and ACH.2 cells. Graph represents relative amounts of p30 ^II ^mRNA in p30 Jurkat T-cells and ACH.2 normalized to β-2 microglobulin.

Our microarray data indicated that p30 modulates a number of genes in T-cells including genes involved in cell cycle and apoptosis control[25]. To examine if p30 expression results in alteration of cell cycle, we infected Jurkat T- cells with a p30 expressing lentivirus and tested the expression of the viral protein by western blot assay (Fig. 1A). p30 mRNA levels were similar between Jurkat T-cells expressing p30 and a primary human lymphocyte derived cell line immortalized by an HTLV-1 full-length proviral clone (ACH.2)[42,43] by reverse transcriptase PCR (Fig. 1B). Typically at least 88 – 92 % of Jurkat T-cells were GFP positive in both p30 and mock Jurkat T-cells by FACS in four trials (data not shown). We then synchronized p30 and mock transduced Jurkat T-cells at the G1/S boundary by hydroxyl urea treatment to test their ability to progress through the cell cycle. After release from arrest, cells were collected at indicated time points and stained with propidium iodide and monitored for their progression through the cell cycle by flow cytometry.

**Figure 2:**
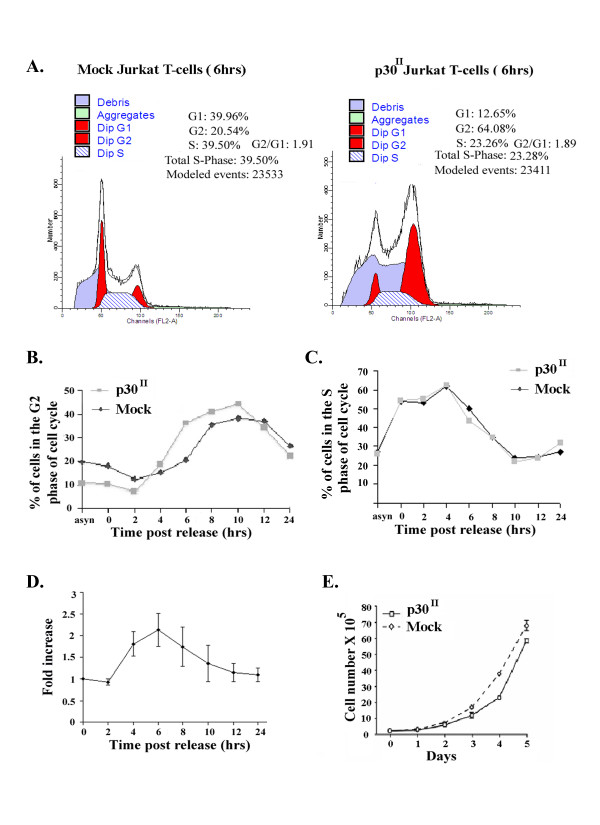
**Progression of p30 expressing Jurkat T-cells through G2-M is delayed**. A) Cell cycle distribution of mock or p30 transduced Jurkat T-cells at 6 h post release from hydroxyl urea block by propidium iodide staining followed by FACS analysis. Histogram was generated using ModFit^R^program (Verity Software House, Topsham, ME). Data represented is from one of the 4 independent trials B) A comparison of percentage of cells in the G2-M phase of the cell cycle of p30 and mock transduced Jurkat T-cells at indicated time points post hydroxyl urea release. Data represented is from a different trial than A. C) A comparison of percentage of cells in the S phase of cell cycle of p30 and mock transduced Jurkat T-cells at indicated time points post hydroxyl urea release. Data represented is from the same trial as B. D) Fold increase of cells in G2 phase of cell cycle in p30 Jurkat T-cells of 4 independent experiments was calculated by dividing the number of cells in G2 phase of cell cycle in p30 Jurkat T-cells over mock Jurkat T-cells. E) p30-expressing Jurkat T-cells or mock infected Jurkat T-cells were assayed for growth using a trypan blue exclusion assay. The p30-expressing Jurkat T-cell line growth curve differed from that of the mock infected Jurkat T-cell line (p-value 0.02 after adjusting for time in a quadratic model) due to an initial lag in the p30-expressing Jurkat T-cell growth rate compared to that of the mock infected Jurkat T-cells. By day 5, p30- expressing Jurkat T cell cultures had fewer total cell numbers compared to mock infected Jurkat T cell cultures (nonparametric Wilcoxon rank sum test, p-value 0.05). Bars indicate 95 % confidence interval.

At 4 h after release, cells started to enter the G2/M phase of cell cycle in both p30 expressing and mock Jurkat T-cells. However, as compared to mock transduced cells, p30 transduced Jurkat T-cells had a higher proportion of cells at the G2/M phase of cell cycle, particularly between 6 to 10 h in 4 independent trails (Fig. 2A and 2B). The observed increase in G2/M population in p30 expressing Jurkat T-cells may be attributed to a faster S phase exit. However, we did not see any significant difference in S phase population between mock or p30 expressing Jurkat T-cells (Fig. 2C). p30 expression resulted in a doubling of the number of Jurkat T-cells in G2 phase of the cell cycle by 6 h after release from synchronization (Fig. 2D). Thus, p30 expression resulted in increased accumulation of cells in the G2/M phase of cell cycle. We hypothesized that if p30 mediated a delay in G2 exit, then the rate at which p30 Jurkat T-cells divide should be different from mock (lentivirus vector lacking p30) transduced Jurkat T-cells. To examine the effect of p30 expression on cell proliferation over an extended time period (1–5 days), we compared viable cell numbers of p30-expressing versus mock infected Jurkat T-cell lines using trypan blue exclusion assay. The number of p30-expressing Jurkat T-cells was significantly reduced compared to mock infected Jurkat T-cells (Fig. 2E). The slower proliferation rate of p30 transduced Jurkat T-cells in these longer term proliferation assays was consistent with the observed G2 cell cycle delay exhibited by p30 expressing cells.

**Figure 3:**
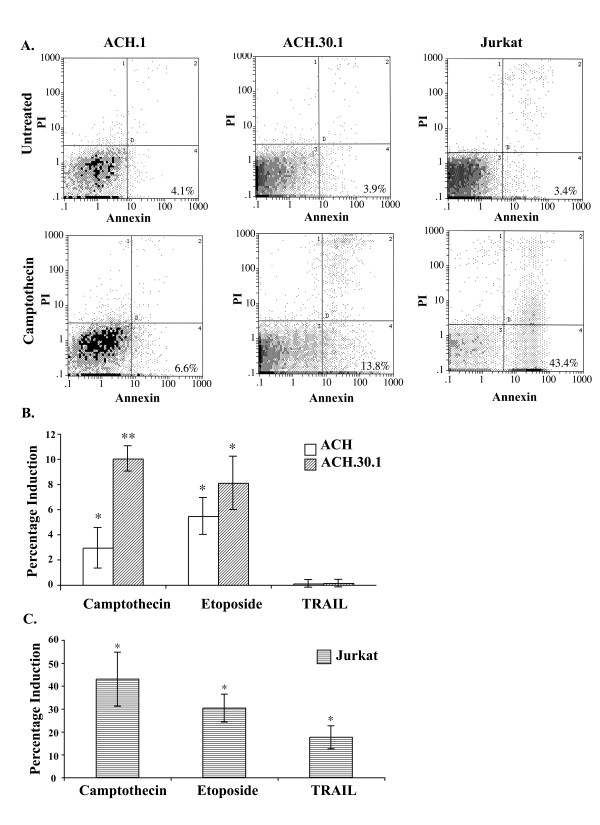
**Differential camptothecin-induced apoptosis in HTLV-1 immortalized cell lines**. A) ACH.1 and ACH.30.1 cell lines were exposed to various apoptosis inducing agents and assayed for percentage of cells induced into apoptosis via Annexin V flow cytometry. Data represents the results of at least three independent experiments. Jurkat T-cells were used as a positive control. Representative result of Annexin V flow cytometry following apoptosis induction with camptothecin. Apoptotic fraction is seen in the lower right quadrant by FACS analysis. B) Following treatment with camptothecin, a greater percentage of ACH.30.1 cells were induced into apoptosis compared to ACH.1 cells (nonparametric Wilcoxon rank sum test, p-value 0.03). ACH.30.1 cells and ACH.1 cells were induced into apoptosis to an equal degree following treatment with etoposide (nonparametric Wilcoxon rank sum test, p-value 0.25). Neither ACH.1 nor ACH.30.1 cells were induced into apoptosis following treatment with TRAIL (nonparametric Wilcoxon rank sum test, p-value 0.59 and 0.41, respectively). C) As a positive control, apoptosis was induced in Jurkat T-cells with all apoptosis inducing agents. * Statistically significant apoptosis induction; ** Statistically more apoptosis induction in ACH.30.1 cells compared to ACH.1 cells following treatment with camptothecin. Standard error bars are indicated.

Adult T-cell leukemia/lymphoma is a highly aggressive CD4+ T-cell malignancy that is refractory to conventional chemotherapeutic intervention [1]. To test the influence of p30 on the ability of T-cells immortalized by HTLV-1 to resist drugs that induce apoptosis, we used cell lines derived from primary human T-cells that were immortalized by wild type HTLV-1 (ACH.1) and a clone of HTLV-1 that is mutated to prevent expression of p30 (ACH.30.1) as previously described [17,42,44]. To determine if the ACH.1 and ACH.30.1 cell lines would display differential sensitivity to apoptotic stimuli, we tested the cell lines following treatment with various apoptosis inducing agents, camptothecin, etoposide, and TRAIL. Camptothecin is a topoisomerase I inhibitor, which induces apoptosis in cells in the S phase of the cell cycle (reviewed in [45]). Etoposide is a topoisomerase II inhibitor, which induces apoptosis via the intrinsic pathway[46,47]. TRAIL is a member of the TNF ligand family, which induces apoptosis through activating the death receptors (reviewed in [48]). In independent trials, camptothecin induced apoptosis in the ACH.30.1 cell line to a greater degree than in the ACH.1 cell line (nonparametric Wilcoxon rank sum test, p-value 0.03) (Fig. 3A). Camptothecin effectively induces apoptosis in cells in the S phase of the cell cycle. This increased susceptibility to camptothecin-induced apoptosis in the ACH.30.1 cell line is likely due to the unabated influence of Tax expression driving cells into the S phase, which would typically be counteracted by p30 [26]. These results are consistent with a recent report [15]. Following treatment with etoposide, there was no significant difference in the degree of apoptosis induction between ACH.1 and ACH.30.1 cell lines (nonparametric Wilcoxon rank sum test, p-value 0.25) (Fig. 3B). Both ACH.1 and ACH.30.1 cell lines lack TRAIL receptor expression and were not susceptible to TRAIL-mediated apoptosis (nonparametric Wilcoxon rank sum test, p-value 0.59 and 0.41, respectively) (Fig. 3B). Jurkat T-cells served as positive control for the apoptotic induction protocols and were susceptible to all treatments (Fig. 3C).

**Figure 4:**
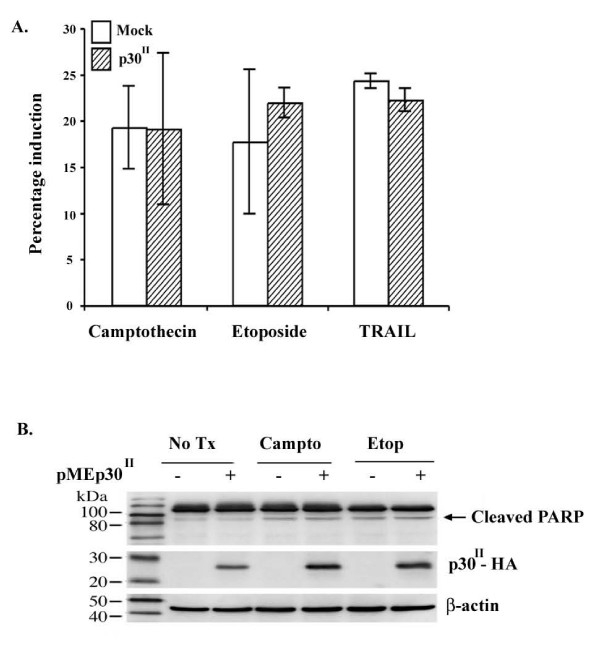
**HTLV-1 p30 does not modulate apoptosis in 293T cells or Jurkat T-cells**. A) p30-expressing Jurkat T-cells or mock infected Jurkat T-cells were treated with camptothecin, etoposide, or TRAIL and assayed for apoptosis induction via Annexin V flow cytometry. Data represents the results of three independent experiments. Although camptothecin, etoposide, and TRAIL induced both cell lines into apoptosis, a differential degree of apoptosis induction was not seen between the two cell lines (nonparametric Wilcoxon rank sum test, p values: camptothecin 0.82, etoposide 0.51, TRAIL 0.13). Standard error bars are indicated. B) 293T cells were transiently transfected with either pME-p30HA or the empty pME-18S vector. Cells were untreated or treated with camptothecin or etoposide. Cell lysates were harvested and 50 μg of lysate was separated by SDS-PAGE. Apoptosis was assayed via immunoblot for the 89 kDa fragment of cleaved PARP. Expression of p30 was verified via immunoblot for HA. Expression of β-actin was verified as a loading control. - cells transfected with empty vector control; + cells transfected with pME-p30HA.

We then tested the influence of exogenously expressed p30 on susceptibility of cells to apoptosis independent of other viral proteins. p30 was transiently expressed in Jurkat T-cells and 292T cells and tested for susceptibility to apoptotic stimuli. Expression of p30 in Jurkat T-cells did not result in increased apoptosis when left untreated, compared to mock infected cells, consistent with recent findings that p30 does not induce apoptosis in transiently transfected Molt-4 lymphocytes[15]. p30-expressing Jurkat T-cells and mock infected Jurkat T-cells were treated with camptothecin, etoposide, or TRAIL and assayed for apoptosis (Fig. 4A). Although the transduced cells were induced into apoptosis following treatment with camptothecin, etoposide, and TRAIL, there was no significant difference in the percentage of apoptotic cells between p30-expressing T-cells and mock Jurkat T-cells for any of the treatment groups (nonparametric Wilcoxon rank sum test, p values: camptothecin 0.82, etoposide 0.51, TRAIL 0.13). To examine the role of p30 in modulating cellular apoptosis in other cell types, we transiently transfected 293T cells with either pME-p30 HA or empty vector control (pME-18S). Following treatment with camptothecin or etoposide, cells were tested for apoptosis using immunoblot assay for the 89 kd fragment of cleaved PARP. Consistent with our data using Jurkat T-cells, we did not observe an increase in susceptibility to apoptosis between p30-expressing cells and negative control cells (Fig. 4B), and lead us to further test the influence of the viral protein in cell cycle regulation.

**Figure 5:**
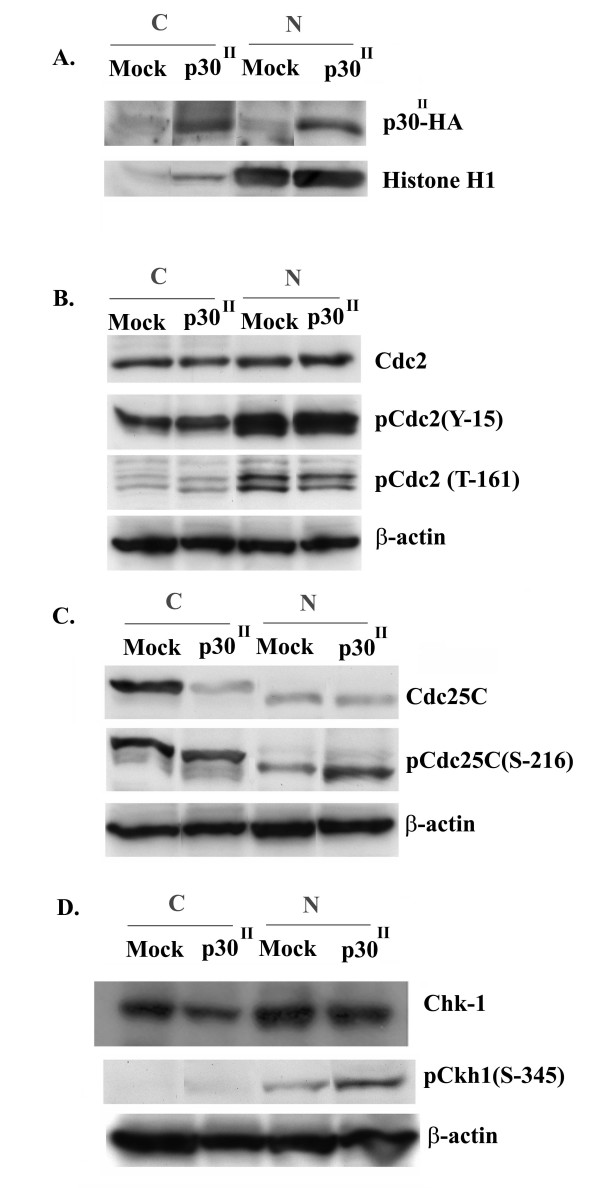
**p30 enhances phosphorylation of Cdc25C at S-216**. A) 40 ug of nuclear (N) or cytosolic (C) fraction prepared from either p30 expressing or Mock Jurkat T-cells were loaded on 10% SDS gel and western blot analysis was performed with anti-HA to confirm p30 expression and Histone H1 western to confirm nuclear and cytosolic fractionation. B) Western blot of nuclear (N) and cytosolic (C) extracts prepared from p30 or mock Jurkat T-cells probed with anti-Cdc2 and phosphospecific Cdc2 antibody. C) Western blot of nuclear (N) and cytosolic (C) extracts prepared from p30 or mock Jurkat T-cells, probed with anti-Cdc25C or phosphospecific anti-Cdc25C. D) Western Blot analysis of nuclear or cytosolic extracts from p30 or mock Jurkat T-cells, probed with anti-Chk1 or monoclonal phosphospecific Chk1 (S-345).

**Figure 6:**
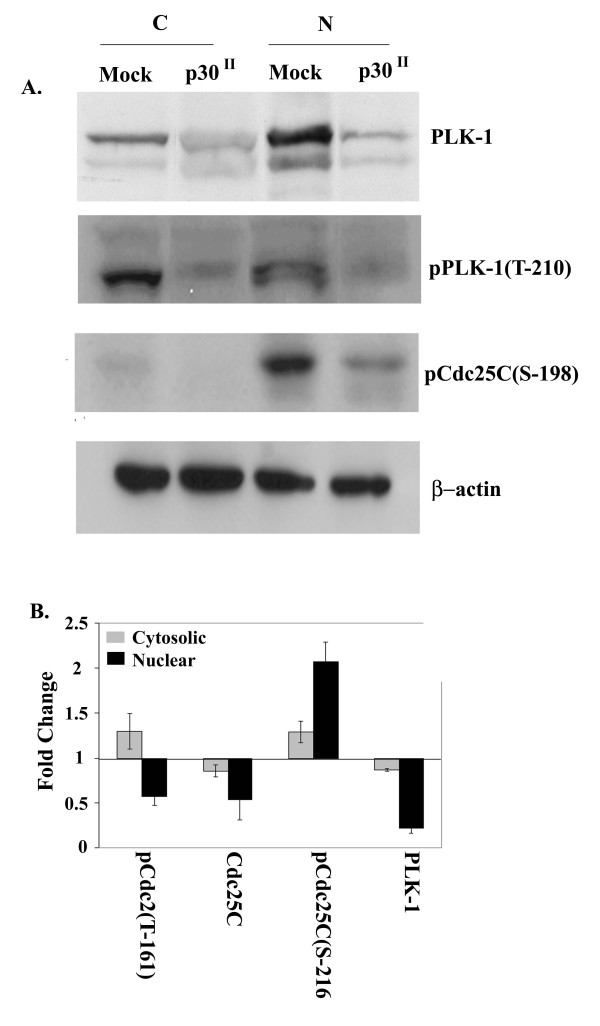
**PLK1 protein level is reduced in p30 Jurkat T-cells and overall quantified comparisons**. A) Western blot analysis of cytosolic (C) or nuclear extract (N) prepared from either p30 expressing or mock Jurkat T-cells and probed with anti-PLK1, pPLK-1(T-210) and pCdc25C(S-198). β-actin was used as a loading control. B) Densitometric analysis of western blot: Band intensity was quantified by Gel-Pro Analyzer 3.1^® ^and normalized to β-actin. Graph represents densitometric analysis of 4 independent western blots for each of the represented proteins.

To further examine p30 mediated G2 delay, we next tested the expression of cyclin B1 and Cdc2 in p30 expressing Jurkat T-cells. During cell cycle progression, the G2-M transition is mediated by active Cdc2 and cyclin B1 complex [49]. Our data indicated that asynchronous Jurkat T-cells expressing p30 had no change in cyclin B1, Cdc2, or phosphorylated Cdc 2 at tyrosine 15, but a 1.5 fold decrease in phosphorylation of Cdc2 at threonine 161 compared to mock infected Jurkat T-cells (Fig. 5B and Fig. 6B). These results lead us to further examined proximal signals of cell cycle regulation that could explain a delay in G2/M transition in p30 expressing T-cells.

The activity of Cdc2 is regulated by the phosphatase Cdc25C. Dephosphorylation of Cdc2 at threonine 14 and tyrosine 15 by Cdc25C results in activation of Cdc2 and initiation of an autoactivation loop between Cdc25C and Cdc2 that efficiently drives cells into mitosis. We reasoned that since p30 expression is associated with a decrease in phosphorylation of Cdc2 at threonine161, we anticipated a less active form of Cdc25C. To test this hypothesis we examined the expression and phosphorylation status of Cdc25C in p30 and mock Jurkat T-cells. No change was observed in the amounts of nuclear Cdc25C in p30 expressing Jurkat T-cells (Fig. 5C) or transcript levels of Cdc25C by reverse transcriptase PCR when compared to mock transduced Jurkat T-cells (data not shown).

We next tested the phosphorylation status of Cdc25C at serine 216 using phosphospecific antibodies by western blot assay. Interestingly, p30 expression resulted in enhanced phosphorylation of Cdc25C at serine 216 and an increase in accumulation of the phosphorylated form in the nucleus in both p30 transduced Jurkat T-cells (Fig. 5C) and 293T cells transfected with pME p30 (data not shown). These data indicate that p30 expression was associated with an increase in nuclear accumulation of Cdc25C phosphorylated at serine 216, consistent with a delay in G2 exit from the cell cycle.

Phosphorylation of Cdc25C at serine 216 is mediated primarily by Chk1 and other kinases including Chk2 or Cdc25C associated kinase (cTAK1). Chk1 is activated by phosphorylation mediated by ataxia telangiectasia mutated and rad 3 related kinase (ATR) in response to single stranded DNA breaks[50]. We therefore examined the phosphophorylation status of Chk1 at serine 345 in p30 expressing and mock infected Jurkat T-cells. Consistent with enhanced phosphorylation of Cdc25C at serine 216 and a delay in G2 exit from the cell cycle, we observed an enhanced phosphorylation of Chk 1 at serine 345 (Fig. 5D and 6B).

Phosphorylation of Cdc25C at serine 198 by PLK1 results in nuclear localization of Cdc25C by eliciting a conformational change that conceals its nuclear export signal [40,41] and therefore PLK-1 has been described as a positive regulator of G2/M transition [51]. Polo-like kinase 1 also phosphorylates cyclin B1 and promotes nuclear accumulation of the cyclin B1-Cdc2 hetero dimmer [52]. Polo-like kinase 1 is activated upon phosphorylation at threonine 210 and serine 137 and phosphorylation at these sites is inhibited upon DNA damage to prevent cells from entering mitosis [53]. We therefore examined if PLK1 protein levels were altered in p30 expressing versus mock Jurkat T-cells. Interestingly, p30 expression resulted in reduced amounts of detectable PLK1 and the threonine 210 phosphorylated form of PLK-1 (Fig. 6A). Finally, we examined the phosphorylation status of Cdc25C at serine 198. Consistent with less PLK1, p30 expression resulted in reduced phosphorylation of Cdc25C at serine 198 (Fig. 6A). These data further supported our observed G2/M delay as PLK1 promotes G2/M transition. Using PLK1 specific primers, we examined the transcript levels of PLK1 in p30 and mock transduced Jurkat T-cells by reverse transcriptase PCR and found that p30 did not result in decrease in PLK1 transcript levels (data not shown). The fold change in expression of key G2/M cell cycle regulatory proteins in p30 expressing Jurkat T-cells is summarized in Fig. 6B.

## Discussion

The ability of HTLV-1 to promote T-cell survival is critical to allow the virus to spread cell-to-cell following infection prior to an active immune response. This permits the virus to establish an infection that is maintained life-long through regulated virus expression and clonal expansion of infected lymphocytes [54]. Multiple studies indicate the importance of HTLV-1 ORF II expression during the course of the natural infection. Infected human subjects exhibit antibody and cytotoxic T cell responses against recombinant proteins or peptides of pX ORF II proteins[13,14] and freshly cultured transformed lymphocytes from HTLV-1 patients express both Tax and p30 [15]. We were the first to demonstrated that pX ORF II encoding p30 is necessary for establishment and maintenance of HTLV-1 infection in a rabbit model [16,17]. In this study, we sought to determine if p30 has a functional role in modulating T-cell survival. Herein, we report that expression of p30 in Jurkat T-cells results in an accumulation of cells in the G2 phase of cell cycle. Expression of the viral protein resulted in an increase in phosphorylation of Cdc25C at serine 216, which was presented in greater amounts in the nucleus of p30 expressing cells. The activated form of Chk1 phosphorylated at serine 345 was increased in p30 expressing Jurkat T-cells concurrent with a decrease in expression of PLK1 and the phospho-tyrosine 210 form of PLK1. Consistent with less PLK1, p30 expression resulted in reduced phosphorylation of Cdc25C at S-198. Interestingly, primary human lymphocyte derived cell lines immortalized by an HTLV-1 proviral clone defective in p30 expression were more susceptible to camptothecin induced apoptosis. Collectively, our data indicate that HTLV-1 p30 expression modulates regulatory cell cycle control in T-cells resulting in accumulation of cells in G2-M phase of cell cycle, which would enhance early viral spread and prolong lymphocyte survival.

The effects of p30 in modulation of the cell cycle contrast to the influence of HTLV-1 Tax on cell cycle regulation. We have recently demonstrated that p30 balances and counteracts the influence of Tax [26]. Tax has been reported to interact directly with Chk-2 resulting in attenuation of DNA damage induced signaling in an ATM/chk2-mediated pathway dependent manner [55]. Our data indicates that p30 results in G2-M delay by enhancing Chk-1 phosphorylation. In response to DNA damage, ATR kinase phosphorylates and activates Chk1 resulting in G2 arrest [50]. Thus, p30 may be involved in a DNA damage/repair signaling pattern, similar to HIV-1 Vpr [56-58]. Our current studies indicate that p30 enhances DNA damage/repair signaling in an ATM dependent manner (manuscript in preparation) and suggest a role in integration allowing DNA repair to take place. Thus, p30 counteracts some of the cellular effects of Tax, which if not regulated, could cause premature cell death by apoptosis or a more rapid oncogenic transformation event, which would be detrimental for long-term viral persistence.

HTLV-1 is the etiologic agent of adult T-cell leukemia/lymphoma a highly aggressive CD4+ T-cell malignancy affecting approximately 1–5 % of HTLV-1-infected individuals after a latent period as long as three decades [1]. Our data has implications in our understanding of how the virus establishes infection and immortalizes T-cells in a manner that results in a relative resistance to drug induced apoptosis. T-cells immortalized with HTLV-1 proviruses lacking p30 expression (ACH.30.1) were more susceptible to camptothecin-induced apoptosis. Camptothecin is a topoisomerase I inhibitor, which induces apoptosis in cells in the S phase of the cell cycle (reviewed in [45]). We have recently demonstrated that p30 balances and counteracts the influence of Tax [26]. Without the dampening influence of p30 on Tax, the ACH.30.1 cells would be predicted to be more in the S phase of the cell cycle and susceptible to drugs such as camptothecin. Thus, p30 effects upon the cell cycle, in particular during the early phase of viral spread in vivo may enhance cell survival and promote cell to cell spread of the infection.

Gene array studies have implicated p30 in the modulation of expression of a variety of cellular genes, including many cell cycle and apoptosis regulatory genes [15,25]. To further test potential mechanisms for our observed p30 mediated G2 delay, we tested the expression status of cyclin B1 and Cdc2 key mediators of the G2-M transition. We did not see a change in cyclin B1 or Cdc2 in either p30 expressing or mock Jurkat T-cells. However, p30 expression was associated with a decrease in phosphorylation at threonine 161 supporting the G2 delay observed in p30 expressing Jurkat T-cells. Cdc2 is activated by Cdc25C that removes phosphate groups from tyrosine 15 and threonine 14 [31]. Activated Cdc2 can further activate Cdc25C [39]. We therefore looked at the expression and phosphorylation status of Cdc25C by western blot analysis. When phosphorylated at serine 216, Cdc25C is typically shuttled out of the nucleus by the cytoplasmic anchor protein complex 14-3-3 and therefore excluded from its substrate [32]. We found that p30 expression resulted in reduced protein levels of Cdc25C. It is possible that p30 may repress Cdc25C expression at the transcriptional or posttranscriptional level. We also observed that p30 expression was associated with enhanced phosphorylation of Cdc25C at serine 216. Interestingly we found an increase in nuclear accumulation of Cdc25C phosphorylated at serine 216, which primarily is localized in the cytoplasm. It is possible that p30 might interfere with nuclear export of the protein and therefore cause an accumulation pCdc25C serine 216 in the nucleus. It is also possible that p30 might result in decreased 14-3-3 expression or directly bind with 14-3-3 and result in lesser amounts of 14-3-3 available for binding to and shuttling of pCdc25C serine 216. Our data indicates that p30 expression results in increased amounts of cellular Chk1 serine 345 phosphorylated forms to accumulate, consistent with the increased phosphorylation of Cdc25C.

Polo-like kinase 1 phosphorylates Cdc25C at serine 198 and allows the nuclear retention of Cdc25C by concealing the nuclear export signal [41]. ATR kinase inactivates PLK1 in response to DNA damage. Our data indicates that p30 expression was associated with decreased PLK1 and its threonine 210 phosphorylated form. Reduced total levels of PLK-1 may be because p30 may modulate PLK-1 expression at the transcriptional or posttranscriptional level or may affect stability of PLK-1 protein. Future studies are directed towards understanding the role of p30 in PLK-1 expression.

Consistent with the less active form of PLK-1, reduced phosphorylation of Cdc25C at serine 198 was associated with p30 expression. We expected that if PLK1 expression is low, we should see more cytosolic form of Cdc25C. However in our studies we find that Cdc25C phosphorylated at serine 216 is increased in the nucleus. These data suggests that p30 may inhibit the nuclear export of Cdc25C similar to its effects on tax/rex mRNA [8,21].

Our data suggests parallels between the function of HTLV-1 p30 and HIV-1 Vpr, which is associated with G2 arrest [56]. The biological significance of this arrest during the natural infection is not well understood but the HIV-1 LTR seems to be more active in the G2 phase, suggesting that G2 arrest confers a favorable cellular environment for efficient transcription of HIV-1 [59]. Vpr induced cell cycle arrest requires ATR kinase for the activation of Chk1 that results in phosphorylation and inactivation of Cdc25C [60]. In this regard it will be important to test HTLV-1 LTR activity during the G2 phase of cell cycle and the viral proteins effect upon proviral integration. HIV-1 Vpr expression may increase Survivin expression during G2/M to regulate cell viability during HIV-1 infection [61]. Similarly, p30 may serve to prolong the survival of HTLV-1 infected cells by up regulating key cellular gene products like Survivin to prevent apoptosis and elimination of HTLV-1 infected cells.

## Conclusion

Overall, our data indicates a role for p30 in modulating cell cycle parameters of T-cells providing new insights how HTLV-1 regulates its cellular environment and balances the effects of Tax, which if unchecked would result in rapid immune elimination of virus producing host cells or cause cell death by apoptosis, both detrimental for viral persistence, a hallmark of the natural infection.

## Methods

### Cell lines

ACH.1 and ACH.30.1 cell lines were obtained from outgrowth of immortalized PBMCs transfected with the ACH or ACH.p30 ^II ^clones, respectively[42,44]. Cells were maintained in RPMI 1640 supplemented with 15 % fetal bovine serum, L-glutamine (30 ug/ml), penicillin (100 μg/mL), streptomycin (100 μg/mL), and recombinant IL-2 (10 U/mL) (complete medium). The 293 cell line is a human embryonic kidney epithelial cell line (catalog number 1573, American Type Culture Collection, Manassas, VA). 293T is a clone of the 293 cell line which stably expresses the simian virus 40 (SV40) T antigen (G. Franchini, National Cancer Institute). 293T cells were maintained in modified Dulbecco's Eagle medium containing 10 % fetal bovine serum and streptomycin (100 ug/mL) and penicillin. Jurkat T-cells (American Type Culture Collection clone E6-1; catalognumberTIB-152) were maintained in RPMI 1640 supplemented with 15 % fetal bovine serum, penicillin (100 μg/mL), streptomycin (100 μg/mL), and L-glutamine (30 ug/mL).

### Lentiviral vectors, plasmids and transduction of Jurkat T-cells

Generation of the pWPT-p30HA-IRES-GFP and pWPT-IRES-GFP lentiviral vectors has been described[25]. The pME-p30HA and pME-18S plasmids have also been described[25]. Production of recombinant lentivirus and transduction of Jurkat T-cells with pWPT-IRES-GFP and pWPT-p30HA-IRES-GFP has been described[25]. Cells were analyzed for p30 expression by western immunoblotting and GFP expression by flow cytometric analysis (FACS).

### Cell cycle analysis

p30 or GFP transduced Jurkat T-cells were synchronized with 2 mM hydroxyl urea (Sigma) for 14 h followed by release for 6 h and a second hydroxy urea block for 16 h. Synchronized cells were released from the block and collected at 0, 2, 4, 6, 8, 10, 12, 24 h. Cells were fixed in 70 % ethanol at kept at 4°C for 14 h. Cells were then washed with PBS and treated with DNAse free RNAse (Roche, Indianapolis, IN) for 30 min in PBS containing 0.1 % triton X-100 at 37°C followed by staining with propidium iodide (Sigma) and analyzed by BD FACS Calibur system ^® ^(BD Biosciences, San Jose, CA).

### Assays to test susceptibility to apoptotic agents

For lymphocyte cell lines (ACH.1, ACH.30.1, Jurkat T cells), apoptosis was induced with camptothecin (10 μM, 4 h)[62], etoposide (12 μg/mL, 6 h)[63], and TRAIL (TNF-related apoptosis inducing ligand) (1 μg/mL, 2 h) [64]. 293T cells were induced into apoptosis with camptothecin (10 μM, 24 h)[65] and etoposide (12 μg/mL, 24 h) [66]. Drug doses were optimized for maximal apoptosis induction prior to each experiment.

### Flow cytometry

ACH.1, ACH.30.1, and Jurkat T cells were prepared for flow cytometry by labeling with Annexin V Alexa Fluor^® ^488 conjugage (Molecular Probes, Eugene, OR) and propidium iodide (PI) (Molecular Probes) or Annexin V AlexaFluor^® ^647 conjugate (Molecular Probes) according to the manufacturer's protocol. In brief, the cells were collected, washed once with PBS, and re-suspended at 1 × 10^6 ^cells/mL in 100 μL of Annexin-binding buffer (Molecular Probes), followed by incubation with 5 μL Annexin V conjugate solution and 1 μL 100 μg/mL PI for 15 min at room temp. After the incubation period, 400 μL of Annexin-binding buffer was added, and samples were kept on ice. The samples were analyzed by flow cytometry (Coulter Epics Elite, Beckman Coulter Inc., Fullerton, CA) and data were analyzed using Coulter Flow Center software (Beckman Coulter Inc.). For each sample, 10,000 gated cells were examined for Annexin V and PI staining, and the percentage of cells in early apoptosis was defined by high Annexin V- and low PI-staining cell population. All Annexin V assays were performed in a minimum of three independent experiments. Nonparametric Wilcoxon rank sum test was used for statistical analysis of significant apoptosis induction and comparison of apoptosis induction between cell lines.

### Transient transfections

Subconfluent 293T cells were transfected with 15 μg of either pME-p30 HA or pME-18s using Superfect ^® ^(Qiagen, Valencia, CA) according to manufacturer's protocol. After 48 h, transfected cells were washed with PBS and lysed with RIPA buffer (150 mM NaCl, 0.01 M sodium pyrophosphate, 10 mM EDTA, 10 mM sodium fluoride, 50 mM Tris (pH 8.0), 0.1% SDS, 12.8 mM deoxycholic acid, 10 % glycerol, 1 % NP-40) containing one complete mini protease inhibitor cocktail tablet (Roche Applied Science, Indianapolis, IN). Cell suspensions were incubated on ice for 20 min and the lysates were centrifuged at 14,000 rpm for 20 min at 4°C. Supernatant was stored at -80°C.

### Western blot assays

Expression of cell cycle regulators were analyzed using nuclear and cytosolic extracts. Briefly, cells were swelled in hypotonic buffer (10 mM HEPES pH 7.9, 1.5 mM MgCl_2_, 10 mM KCl) followed by shearing with a 27 gauge needle followed by centrifugation at 14000 rpm for 15 sec. Supernatant were saved as cytosolic fractions and nuclear pellets were incubated with high salt buffer for 1 h (20 mM HEPES pH 7.9, 25 % glycerol, 1.5 mM MgCl_2_, 1.2 M KCl, 0.2 mM EDTA) followed by low salt buffer (20 mM HEPES pH 7.9, 25 % glycerol, 1.5 M MgCl_2_, 0.02 M KCl, 0.2 mM EDTA) and centrifuged at 14000 rpm for 30 min to get nuclear extract. Membranes were blocked with 5 % non-fat dry milk and 10 % fetal bovine serum in Tris-buffered saline with 0.1 % Tween (TBST) for 2 h at room temp., then incubated with primary antibody overnight at 4°C. Immunodetection was performed using the following antibodies: mouse anti-HA monoclonal antibody clone 16B-12 (1:1000, Covance Research Products, Princeton, NJ), mouse monoclonal anti-PARP clone C-2-10 (1:1000, Oncogene Research Products, Boston, MA). mouse monoclonal anti-β-actin clone AC-74 (1:4000, Sigma), rabbit polyclonal anti-Cdc2 # 9112 (1:1000, Cell Signaling, Beverly, MA), rabbit polyclonal anti-PLK1pT210 # 600-401-466 (1: 1000, Rockland, Gilbertsville, PA), polyclonal rabbit anti-Cdc2 T-161 #9114 (1:1000, Cell Signaling), polyclonal rabbit anti Cdc2 Y-15 #9111(1:1000, Cell Signaling), polyclonal rabbit anti-Cdc25C #9522 (1:1000, Cell Signaling), polyclonal rabbit anti-Cdc25C S-216 # 9528(1:1000, Cell Signaling), rabbit polyclonal anti-Cdc25C pS-198 # 9529 (1: 1000, Cell Signaling), polyclonal rabbit anti-Chk1 #2345 (1:1000, Cell Signaling), polyclonal rabbit anti-Chk1 S-345 #2341 (1: 1000, Cell Signaling), rabbit polyclonal anti-PLK1 (1: 1000) (Abcam, Cambridge, MA), mouse monoclonal anti-Histone H1, clone AE-4 (1: 1000, Upstate). Western blots were developed with horseradish peroxidase-labeled secondary antibody (1:1000) and enhanced chemiluminescence reagent (Cell Signaling Technology). All western blots were repeated at least four times.

### Reverse transcriptase PCR

RNA was isolated from p30 Jurkat T-cells and ACH.2 cells using RNAqueous™(Ambion, Auatin, TX)). Two step RT-PCR was performed by using random primers to prepare cDNA followed by PCR using specific primers for indicated genes. p30 primers were used as described[25]. For β-2 microglobulin the following primer set (Invitrogen) was used F5-ACCCCCACTGAAAAAGATAC-3 and R5-ATCTTCAAACCTCCATGATG-3. Cycles were varied from 15 cycles to 30 cycles in order to compare transcript levels between p30 Jurkat T-cells and ACH.2 cells. β-2 microglobulin was used as a control to compare expression levels. PCR products were run on an agarose gel and stained with ethidium bromide and p30 band was quantified and normalized to β-2 microglobulin band using Alphamager^® ^3.24.

## Abbreviations

Check point kinase, Chk

Arbitrary light units, ALU

Polo-like kinase, PLK

Fetal bovine serum, FBS

Human T-lymphotropic virus type 1, HTLV-1

Adult T cell leukemia/lymphoma, ATL

## Competing interests

The authors have no competing financial or other interests involved in the data collection, methods, or in the writing of this manuscript.

## Authors' contributions

Antara Datta, Lee Silverman, Andrew Phipps, Hajime Hiraragi, Lee Ratner, and Michael D. Lairmore have all met the definition of author as outlined by the Retrovirology journal. All authors read and approved the final manuscript. Each has made substantive intellectual contributions to study. Each author has made substantial contributions to conception and design, or acquisition of data, or analysis and interpretation of data. Each author has given final approval of the version to be published. Each author has participated sufficiently in the work to take public responsibility for appropriate portions of the content.
